# Postpartum recovery after severe maternal morbidity in Kilifi, Kenya: a grounded theory of recovery trajectories beyond 42 days

**DOI:** 10.1136/bmjgh-2023-014821

**Published:** 2024-06-25

**Authors:** Ursula Gazeley, Marvine Caren Ochieng, Onesmus Wanje, Angela Koech Etyang, Grace Mwashigadi, Nathan Barreh, Alice Mnyazi Kombo, Mwanajuma Bakari, Grace Maitha, Sergio A Silverio, Marleen Temmerman, Laura Magee, Peter von Dadelszen, Veronique Filippi

**Affiliations:** 1 Department of Infectious Disease Epidemiology, London School of Hygiene & Tropical Medicine, London, UK; 2 Department of Population Health, London School of Hygiene & Tropical Medicine, London, UK; 3 Centre of Excellence in Women and Child Health, Aga Khan University, Nairobi, Kenya; 4 Department of Obstetrics and Gynaecology, Aga Khan University, Nairobi, Kenya; 5 Department of Women and Children's Health, King's College London, London, UK; 6 School of Psychology, Faculty of Health, Liverpool John Moores University, Liverpool, UK; 7 Institute of Women and Children's Health, King's College London, London, UK

**Keywords:** maternal health, mental health & psychiatry, global health, qualitative study

## Abstract

**Introduction:**

The burden of severe maternal morbidity is highest in sub-Saharan Africa, and its relative contribution to maternal (ill) health may increase as maternal mortality continues to fall. Women’s perspective of their long-term recovery following severe morbidity beyond the standard 42-day postpartum period remains largely unexplored.

**Methods:**

This woman-centred, grounded theory study was nested within the Pregnancy Care Integrating Translational Science Everywhere (PRECISE) study in Kilifi, Kenya. Purposive and theoretical sampling was used to recruit 20 women who experienced either a maternal near-miss event (n=11), potentially life-threatening condition (n=6) or no severe morbidity (n=3). Women were purposively selected between 6 and 36 months post partum at the time of interview to compare recovery trajectories. Using a constant comparative approach of line-by-line open codes, focused codes, super-categories and themes, we developed testable hypotheses of women’s postpartum recovery trajectories after severe maternal morbidity.

**Results:**

Grounded in women’s accounts of their lived experience, we identify three phases of recovery following severe maternal morbidity: ‘loss’, ‘transition’ and ‘adaptation to a new normal’. These themes are supported by multiple, overlapping super-categories: loss of understanding of own health, functioning and autonomy; transition in women’s identity and relationships; and adaptation to a new physical, psychosocial and economic state. This recovery process is multidimensional, potentially cyclical and extends far beyond the standard 42-day postpartum period.

**Conclusion:**

Women’s complex needs following severe maternal morbidity require a reconceptualisation of postpartum recovery as extending far beyond the standard 42-day postpartum period. Women’s accounts expose major deficiencies in the provision of postpartum and mental healthcare. Improved postpartum care provision at the primary healthcare level, with reach extended through community health workers, is essential to identify and treat chronic mental or physical health problems following severe maternal morbidity.

**WHAT IS ALREADY KNOWN ON THIS TOPIC**

- The burden of severe maternal morbidity on women, their families and health systems is significant.
- Severe maternal morbidity increases the likelihood that women experience long-term adverse physical, mental, economic and social outcomes.

**WHAT THIS STUDY ADDS**

- We develop a grounded theory of women’s recovery following severe maternal morbidity based on women’s accounts of their experience, represented by three themes of ‘loss’, ‘transition’ and ‘adaptation to a new normal’.
- This recovery is (1) multidimensional, affecting women’s physical, mental, social, sexual and economic well-being; (2) cyclical across the female reproductive life course, from repeat pregnancies and recurrent episodes of maternal morbidity; and (3) protracted far beyond 42 days post partum.

**HOW THIS STUDY MIGHT AFFECT RESEARCH, PRACTICE OR POLICY**

- Women’s long-term, complex needs after severe maternal morbidity demand a reconceptualisation of the postpartum period of recovery as continuing far beyond 42 days after childbirth.
- Women’s postpartum experience after severe maternal morbidity highlights the need for better communication from healthcare workers about the morbidity event that women experienced; improved access to postpartum and mental healthcare at the primary healthcare level; and specialist care for chronic problems in the extended postpartum period.

## Introduction

There is growing recognition that the focus of the global maternal health community should expand beyond survival.^1^ For every woman who dies from a maternity-related cause, maternal morbidity affects many more women, their families, communities and health systems.^2^ As countries advance through the obstetric transition^3^—where maternal mortality declines and shifts from direct obstetric to indirect causes—the relative contribution of maternal morbidity to maternal ill health will continue to increase.

Adopting WHO terminology, severe maternal morbidity includes potentially life-threatening conditions (PLTCs) and maternal near-miss (MNM) events—life-threatening complications so severe that women would probably have died without receiving emergency obstetric care.^4^ Severe maternal morbidity may occur before, during or after birth, and recovery post partum is physical, emotional, sexual, social and economic in nature. Although ‘postpartum’ is defined as the 42 days after birth, during which time the WHO recommends routine clinical contacts,^5^ some women will not fully recover within this time frame. Compared to women with uncomplicated pregnancies, women who experience severe morbidity are more likely to experience adverse outcomes in the years following birth, such as chronic hypertension^6^ or mental health problems,^7^ economic hardship and sexual violence,^8^ impaired functioning,^9^ lower quality of life^10^ and elevated mortality.^11^ These women’s needs may be diverse and context specific, requiring tailored packages of support after hospital discharge.^12^

There is a dearth of women-centred, in-depth research which seeks to understand women’s lived experience of postpartum recovery beyond 42 days. An exclusive focus on clinical and biomedical aspects of postpartum recovery overlooks women’s personal, interpersonal and cultural interpretation of their morbidity.^13 14^ It is vital to understand the diverse ways that women may experience recovery from severe pregnancy-related morbidity, and how their experiences are shaped by individual, cultural and societal influences.^14^

This study aims to develop a grounded theory of women’s experience of postpartum recovery trajectories after severe maternal morbidity in Kilifi County, Kenya—a theory which can be tested in other contexts and among different populations.

## Methods

The Consolidated Criteria for Reporting Qualitative Research checklist^15^ guided the reporting of our methods (see online supplemental table S2).

10.1136/bmjgh-2023-014821.supp1 Supplementary data

### Study design

Straussian grounded theory methodology, rooted in interpretivist ontology and epistemological contextualism,^16^ was used to generate a new theory about women’s recovery trajectories after severe maternal morbidity. Grounded theory is ideal for use when the evidence base is limited,^17^ and it is an increasingly popular methodology for cross-disciplinary research on women’s health.^13 18^

### Setting

The study was conducted in Kilifi County, Kenya, which has a high burden of maternal morbidity, with facility-based MNM ratio estimates of 7.2^19^–10.4^20^ per 1000 live births. In Kilifi, >80% of women deliver in a health facility; 83% of mothers receive a postpartum check during the first 2 days after birth; >50% have not been employed in the previous 12 months; >50% do not have drinking water on their premises; and only 12% of households have any form of health insurance.^21^ Antepartum, intrapartum and postpartum care is provided through a government scheme (‘Linda Mama’) for women without health insurance. De jure postpartum services included in this scheme include analgesics, vitamin supplementation, family planning, sexually transmitted infection (STI) testing, insecticidal net provision, and treatment or referral for complications.^22^

This qualitative research was nested within the Pregnancy Care Integrating Translational Science Everywhere (PRECISE) multidisciplinary prospective cohort study in Kenya, designed to phenotype pregnancies in women with placental disorders in sub-Saharan Africa.^23 24^ The PRECISE participating facilities in Kilifi County are Mariakani (semiurban) and Rabai (rural) Sub-County Hospitals. Both facilities now offer Comprehensive Emergency Obstetric and Newborn Care services, although Rabai offered only Basic Emergency Obstetric Care before 2022. Neither hospital has maternal intensive or high-dependency care units. Women requiring higher level care may be referred to the Kilifi County Teaching & Referral Hospital, if ambulances are available.

### Participants

Three groups of participants (n=20) who were at least 6 months post partum were purposively selected, including women who experienced an MNM event (n=11); women who experienced a PLTC (n=6); and women who had no severe morbidity (n=3). The study timelines of the PRECISE cohort, which began enrolling women in November 2019, set the upper limit on how many months postpartum participants were at the time of interview. Purposive sampling was used to include women from diverse backgrounds (education, age, parity, type of morbidity, live birth/perinatal death, child health) and at different postpartum stages. Since the postpartum experiences of women who had experienced either an MNM or a PLTC were the primary research question, we kept the numbers of women with no maternal morbidity deliberately small. However, the women with no maternal morbidity were selected to reveal women’s recovery trajectories in the absence of severe morbidity, to understand whether women experience commonalities in their postpartum recovery, despite morbidity.

MNM was defined by WHO standard criteria of organ dysfunction,^4^ or modified criteria for low-resource settings (ie, Haydom^25^ and Tura^26 27^ criteria), as many of the WHO criteria were not applicable in the study sites. These modifications include some severe conditions and interventions as sufficient for MNM. PLTC was defined by WHO criteria^4^ (for detailed definitions of MNM and PLTCs, see online supplemental tables S3 and S4). Participant recruitment was iterative to explore emerging codes, super-categories and themes which required further validation (ie, theoretical sampling).

Table 1 presents participant characteristics for the 20 women interviewed.

**Table 1:** Participants’ background characteristics

Participant code	Age group at enrolment	Relationship status at enrolment	Occupation at enrolment	Parity at enrolment	MNM criteria* met	Morbidity, intervention and organ dysfunction (WHO only)	Delivery type	Months between childbirth and interview
MNM and neonatal death								
Woman 1	40–44	Married	Market trader	Nulliparous	WHO, Haydom and Tura	Severe pre-eclampsia; hepatic dysfunction	Caesarean	24
Woman 2	20–24	Single	Professional services	Nulliparous	Tura only	Severe sepsis	Unassisted vaginal without episiotomy	16
MNM and live birth								
Woman 3	15–19	Cohabiting	Housewife	Nulliparous	Haydom and Tura	Severe PPH; RBC transfusion	Caesarean	6
Woman 4	25–29	Married	Professional services	1	WHO, Haydom and Tura	Severe pre-eclampsia; hepatic dysfunction	Unassisted vaginal without episiotomy	24
Woman 5	20–24	Single	Student	Nulliparous	WHO, Haydom and Tura	Eclampsia; hepatic and neurological dysfunction	Caesarean	24
Woman 6	30–34	Married	Housewife	3	Tura only	Severe pre-eclampsia and pulmonary oedema	Unassisted vaginal with episiotomy	30
Woman 7	35–39	Married	Factory worker	4	Haydom and Tura	Eclampsia	Caesarean	24
Woman 8	30–34	Married	Small business owner	3	Haydom and Tura	Severe sepsis	Unknown	28
Woman 9	25–29	Married	Casual labourer	1	WHO, Haydom and Tura	Severe pre-eclampsia; haematological dysfunction	Unassisted vaginal without episiotomy	5
Woman 10†	35–39	Married	Market trader	4	WHO, Haydom and Tura	Blood transfusion; respiratory dysfunction	Caesarean	15
Woman 11‡	30–34	Married	Housewife	2	N/A	Retained placenta; PPH; emergency referral	Unassisted vaginal without episiotomy	36
Woman 12	25–29	Married	Housewife	10	Haydom and Tura	Ruptured uterus	Unassisted vaginal without episiotomy	36
Woman 13	25–29	Married (polygamous)	Housewife	2	Tura only	Severe pre-eclampsia; laparotomy	Unassisted vaginal without episiotomy	15
Woman 14	30–34	Married	Retailer	1	Haydom only	Severe PPH; RBC transfusion; laparotomy	Unassisted vaginal with episiotomy	10
PLTC and stillbirth								
Woman 15	35–39	Single	Housewife	1	N/A	Severe pre-eclampsia	Caesarean	6
PLTC and live birth								
Woman 16	15–19	Married	Housewife	Nulliparous	N/A	Severe PPH	Unassisted vaginal without episiotomy	20
Woman 17	30–34	Married (polygamous)	Housewife	Nulliparous	N/A	Severe pre-eclampsia	Unassisted vaginal without episiotomy	13
No severe morbidity								
Woman 18	15–19	Single	Student	Nulliparous	N/A	N/A	Unassisted vaginal without episiotomy	17
Woman 19	25–29	Married	Professional services	Nulliparous	N/A	N/A	Unassisted vaginal without episiotomy	16
Woman 20	30–34	Married	Housewife	2	N/A	N/A	Unassisted vaginal without episiotomy	14

*Further detail can be found in online supplemental tables S3 and S4. For WHO criteria, please refer to Say *et al* 4; for Tura criteria for sub-Saharan Africa, please refer to Tura *et al* (2017); for Haydom criteria, please refer to Nelissen *et al*.^26^

†For this participant only, we identified child developmental problems—measured using the Malawi Developmental Assessment Tool (MDAT).

‡This participant’s medical records were mostly missing because she delivered when PRECISE data collection was suspended due to COVID-19 regulations in 2020. During her interview, she described experiencing a retained placenta, severe haemorrhage, loss of consciousness and an emergency referral to Mariakani Hospital. She was admitted to hospital for 4 days, which was longer than most women in our sample. We therefore considered this participant in the MNM group.

MNM, maternal near miss; PLTC, potentially life-threatening condition; PPH, Postpartum haemorrhage; PRECISE, Pregnancy Care Integrating Translational Science Everywhere; RBC, Red blood cell.

### Materials

The research team collectively reviewed and modified the semistructured interview guide in both English and Swahili, to ensure questions were accurately conveyed and culturally appropriate. The English interview guide is available in online supplemental table S3.

### Data collection

Eligible participants were first invited to participate in the study, by telephone, by a PRECISE community engagement team member, with whom they were familiar. In person, before participation, interested individuals received detailed written and verbal information, which emphasised that participation is voluntary, they have a right to withdraw, to confidentiality, and to data anonymisation. One individual declined due to scheduling conflicts. All participants provided written informed consent.

Face-to-face interviews were conducted in Swahili (April to May 2023), by clinical and non-clinical, experienced qualitative researchers (MCO, NB, MB, AMK, GMa). Best practices for sensitive interviews were followed,^28^ including a choice of private interview locations, conducted away from their partner. All participants chose to be interviewed at the facility. Some women brought their infants to the interview, to breastfeed or because of childcare constraints. 20 interviews were conducted, each lasting 35–70 minutes. There were no repeat interviews. Theoretical saturation was considered to have been reached when new data no longer provided additional insights, so that the resulting theory was adequately grounded in the data.^29^

Women were 6–36 months post partum at the time of interview to facilitate an analysis of recovery trajectories. Participants with ongoing physical or psychological problems were offered referral and counselling information. Five attended a group session with a psychotherapist following, but not as a result of, the interview; no women took up the offer of referral. Participants were reimbursed for transport costs and refreshments were provided. To support research staff, team members received two group debriefs with a trained psychotherapist during the data collection period.^28^

### Data analysis and interpretation

With consent, interviews were audio recorded and transcribed verbatim in Swahili to preserve non-lexical elements capturing participants’ emotions when talking. Transcripts translated into English were reviewed by bilingual team members (MCO, OW) for accuracy.

We followed an established approach to conducting grounded theory analysis^18^ using inductive, ‘bottom-up’ coding derived directly from the data. First, transcripts were ‘open coded’ by hand to capture the phrase or word which best reflected each line of discourse. ‘Open codes’ were then grouped into focused codes applied to multiple sentences or whole paragraphs. Through a process of constant comparative analysis within and between transcripts and fieldnotes,^30^ focused codes were aggregated into super-categories, and finally into themes. Relationships between themes were used to generate a grounded theory—a testable hypothesis derived from and grounded in the qualitative data. Data interpretation was an iterative exercise within the research team which continued until we reached consensus.

The lead researcher (UG) coded and analysed all transcripts. Two members of the research team (MCO, OW) coded and analysed a subset (20%) of the transcripts chosen purposively. When differences in interpretation arose, those of Kenyan researchers were prioritised, given their greater understanding of local context, including cultural customs in Kilifi.

### Positionality statement

Our research team is multinational and multidisciplinary: of 14 coauthors, 9 are based in Kilifi, 8 are Kenyan nationals and 6 have a clinical background (further detail is available in our Reflexivity Statement in the online supplemental file 2). As a combination of cultural insiders and outsiders, we were able to leverage both groups’ strengths in conducting qualitative research, with adequate scrutiny of potential insider-outsider biases.^31^ Each interviewer kept fieldnotes to document their experience of the interview, including participants’ emotional responses to interview prompts. These memos were triangulated with the transcripts to better capture the full contextual picture of participants’ responses. We adopted a relative approach to reflexive judgement, considering how our perspectives and biases (subjectivity) are relative and historically situated within the broader social, cultural and contextual factors of the study. This recognises reflexivity as a continuous process and was implemented through cross-team discussions during tool design, data collection, analyses and interpretation.

10.1136/bmjgh-2023-014821.supp2 Supplementary data

## Results

The following three themes emerged from interconnected super-categories and represent women’s experience of recovery following severe maternal morbidity throughout the extended postpartum period:

1. Loss: (a) of understanding, (b) functioning and (c) autonomy.
2. Transition: (a) in identity and (b) relationships.
3. Adaptation: (a) physical, (b) psychosocial and (c) economic recovery beyond 42 days.

**Figure 1:**
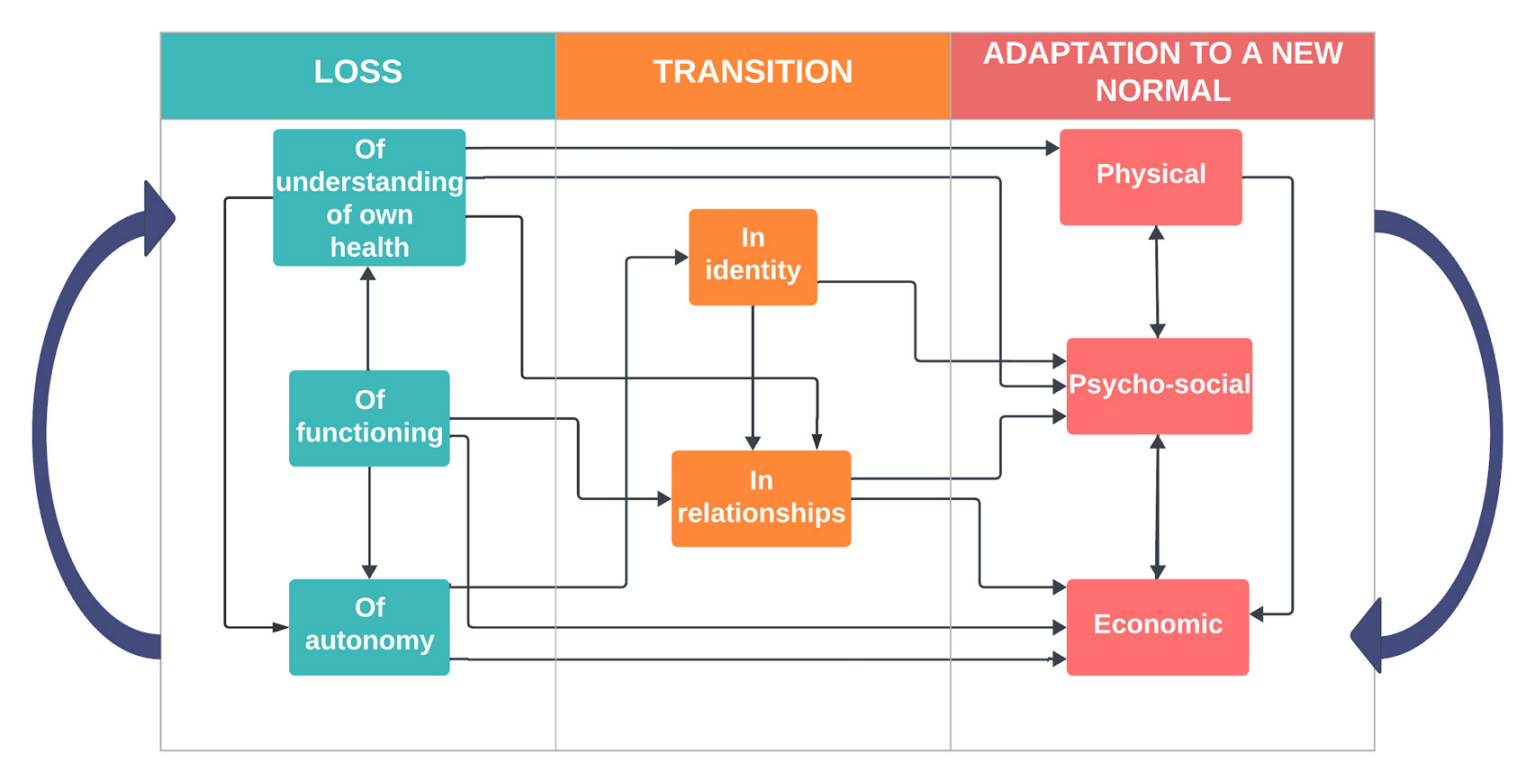
Conceptual mapping of grounded theory: ‘Loss’, ‘transition’ and ‘adaptation to a new normal’.

Figure 1 presents the conceptual mapping of these themes and super-categories and illustrates how recovery may be non-linear and cyclical (in the case of repeat pregnancy).

Illustrative quotations are found by way of analysis below, but additional supporting quotations for refined super-categories and themes can be found in online supplemental table S5.

### Theme 1: loss

Women described multifaceted dimensions of ‘loss’ which incorporated both biomedical dimensions of a MNM or PLTC (ie, loss of blood or consciousness, or their baby’s death), but also psychosocial dimensions (ie, loss of understanding of her health, functioning or autonomy).

#### Loss of understanding (of her own health)

Women’s comprehension about the causes, consequences and treatment of their PLTC or MNM event varied greatly. While some women received and understood explanations from doctors, nurses or midwives, others received explanations they did not understand, and many women did not recall receiving any explanation from providers involved in their care. Lack of understanding affected women’s ability to process their experience, compounded trauma and silenced their experience. For some women, this exacerbated confusion over whether postpartum symptoms were ‘normal’, and eroded trust in the healthcare services. The need to improve providers’ health communication emerged as a key recommendation for improved quality of care:

They didn’t tell me anything. They said you are badly torn inside, I heard that only reaching the theatre. I couldn’t tell how I got there. I don't know what happened… I would like to ask because now I can talk and say ‘mmmh what was done to me, was it surgery or what?’ I mean I don’t know… I gave birth in the hospital, but I can’t really tell what happened. That’s the area to be improved, like they should at least write a report so that even if they won’t tell the patient, whoever comes in the morning can read the report. Like in my case, whenever I touched inside I could only feel strings but I didn’t know anything and I didn’t know who I could ask. (Woman 12)

Women whose severe morbidity coincided with stillbirth or neonatal death also received poor communication, particularly around the cause of their babies’ death:

Upon giving birth, they never explained what had transpired, I was just discharged. I went home, and I haven’t been called. (Woman 2)

However, for one woman whose baby died, her feelings towards understanding the biomedical causes were more complicated, and intertwined with spiritualism and fatalism:

Aaa I don’t want to know [what happened] because that won’t bring back my baby to the world. The baby did not die in anyone’s stomach, he died in my own stomach, and God himself is the one who knows why he took the child. Neither me nor the doctor knows. (Woman 15)

For some women, the severe morbidity they experienced coincided with a loss of consciousness or distorted memory. In these cases, the channel of communication first between the health worker and birth companion, and later between companion and women themselves, was critical for women to understand what happened. It was common in Kilifi for women to be accompanied to the facility by a female family member (typically her mother or mother-in-law), rather than her partner. However, a few women saw the absence of their partner as key to their poor understanding, and held him accountable:

I was told he [the doctor] used to explain but there was a time I had lost my memory…I left crying and told my partner ‘I went through a difficult situation and I escaped death, but you [her partner] failed to explain to me.’ (Woman 7)

It bothers me because I don’t know what procedures were done to me. You see and I insist a lot, if my partner would have been there, then he’s have known if his wife had been taken to theatre and known what was done to her. (Woman 12)

#### Loss of functioning

All women in our sample experienced a loss of functioning across multiple physical and psychosocial domains during the (extended) postpartum period which compromised their ability to carry out daily activities. This was typically more protracted for women with severe morbidity, and in some instances impaired their ability to care for their new baby:

I cried… because I asked myself which situation is this? The child is required to be carried, me myself I cannot sit well…just give me strength God remove all these difficult situations so that I can at least carry my child. (Woman 13)

Loss of physical and cognitive functioning was at times profound enough that women experienced dissociation from their body:

I was feeling a lot of pain… I didn’t understand my body. I felt I couldn’t do anything. (Woman 3)

A loss of functioning at times caused social isolation in the extended postpartum period:

I couldn’t meet up with people. I used to lock myself up in the house, because you can go to that place but maybe it is an activity now you cannot do, so I wasn’t getting out of the house. (Woman 14)

An inability to carry out daily tasks was a cause of anxiety for women and their partners. While some partners responded to women’s loss of functioning with love, empathy and a willingness to help, it was common for women to express sadness and disappointment at their partners’ response. In some relationships, this led to conflict:

He told me ‘You should go and help mother’, I would tell him ‘Aaa right now I don’t have strength to help mother, it will reach a time that I will help her’ so he was just getting angry. (Woman 14)

With only a few exceptions, it was women’s mother or mother-in-law who played the most substantial role in the provision of household and personal care during the (extended) postpartum period, often for many months. This reflects gendered expectations of support deemed to be a woman’s role, but also practical considerations—with many partners working away from home (eg, in Mombasa), and perceived benefits of learning from mothers’ first-hand experience of pregnancy and postpartum recovery:

She [her mother-in-law] supported me well. She used to take care of the baby. As a first-time mother I didn’t know how to bathe a baby, but she did all that for me. (Woman 19)

Women’s and/or their families’ expectations of partners post partum primarily related to financial support. However, in a few cases, especially where maternal support was unavailable (mother was deceased or living far away), partners were more closely involved in the provision of household and personal care. Conversely, for single mothers or where the event precipitated the breakdown of the relationship, refusal of the baby’s father to provide financially for the baby meant their own mothers often assumed roles as both caregiver and financial provider:

After delivery I had broken up with my partner. My mother was the one helping me with pampers, everything that the baby needed my mother was the one providing, everything—pampers, clothing, basins, and everything. She used to support in food also washing the baby and looking after me. (Woman 9)

#### Loss of autonomy

A loss of functioning affected women’s autonomy to choose where, and from whom, she received care. For some, this meant living with their mother or mother-in-law for several months, at times, against her wishes. While this postpartum loss of autonomy is evident among all women in our sample, it affected women with severe morbidity more acutely, as she was expected to stay away to recover for longer:

I was upset because they forced me to go there [to the mother-in-law’s] and there was no way I was going to stay with them. He [the participant’s partner] forced me to go there… When the child started to sit, I came back to my place. (Woman 16)

For some women who were in paid work, pregnancy initiated a loss of financial autonomy, particularly for women working in the informal sector without maternity leave entitlement. Disruption in formal employment was typically more protracted for women with severe morbidity:

[Before the event] I was working and my partner was working too. I was used to catering to my own needs, so it was a challenge because I was not used to depending on him for everything. It was difficult for me to ask him for the children’s needs… Sometimes I was even afraid to ask him because I thought he had no money. (Woman 7)

### Theme 2: transition

#### In identity

As part of a normal postpartum course, pregnancy precipitated complex transitions in women’s identity. For younger mothers, the birth of a child signified her own transition from childhood to adulthood. Many women expressed happiness at their transition to motherhood (for nulliparous women) or to a larger family size (for parous women):

I am just happy when I see my baby. Honestly the baby is my source of happiness… I feel at peace with my baby. (Woman 16)

Particularly after earlier pregnancy losses, the birth of a first child fulfilled some families’ perceptions that the woman had finally become a ‘true’ wife.

When they [her partner’s family] heard I lost the first pregnancy they started to say, ‘she is not a wife, she is just playing, she is playing with your mind’ so when I got this one [first live birth] even if it really hurt me they themselves told me ‘Now come home we know now that you are a wife.’ (Woman 14)

For women whose child died, and who had no surviving children, the loss led to complicated feelings towards motherhood and their identity as a woman:

Among mothers I was being referred to as a mother, but I felt like my womanhood wasn’t complete without a child. (Woman 1)

#### In relationships

Severe maternal morbidity initiated multifaceted transitions in women’s relationships, especially with their partner. For many women, it affected their desire for sexual intimacy in the extended postpartum period:

I was afraid I was going to get pregnant again, and afraid of seeing death come back. (Woman 6)

Some women preferred delaying sexual intimacy for emotional and physical healing post partum. While some partners were understanding, others were not, and one resorted to coercive behaviour to resume sex:

‘If affected him because he wanted us to do the act of marriage and I couldn’t because I was worried he can make those stitches tear, when they open I would have to be stitched again, or he can make us get another baby when this child is still young.’ [After how long did he ask you?] ‘Just two weeks’ [Okay and how did he feel?] ‘He got very angry… he got angry until he said, ‘I will marry another woman.’ (Woman 14)

The experience of severe maternal morbidity also affected women and their partners’ plans for their future family size, although the impact on fertility intentions was sometimes discordant within couples. Motivated by a need to regain strength after the event, many women expressed a desire to space or postpone future childbearing:

I should take a rest. I am not thinking about having another baby until this baby reaches the 5 years, then I will see. [Why?] The pain I felt. (Woman 11)

Some women expressed a desire to stop childbearing altogether:

I said this should be the last born, I won’t give birth again. Because of economics and the problem [morbidity] I went through. (Woman 8)

As witnesses to the event and their wife’s long postpartum recovery, some partners changed their attitudes towards future pregnancies:

I am not worried. The worries were left to my partner maybe, to me I did not have any problem. When he saw the report about the pressure, and when he remembered the previous miscarriage, let’s say the truth is it hurt him mentally…he said he did not wish us to have another baby. (Woman 4)

For two nulliparous women whose child did not survive, anxiety around childbearing differed depending on whether she conceived again – fear that the baby who died was the only one they would ever have, or fear that their next baby would also die:

Maybe I have the problem that I was privileged with that one child, we won’t know where the problem is. Pressure is on both sides [to conceive again]. (Woman 2)

When I got pregnant for the second time, I was afraid… will my child live or die. (Woman 1)

Finally, the impact of severe maternal morbidity on the stability of women’s relationships differed depending on whether their baby survived. For some couples with a surviving baby, the baby was a source of joy that helped redefine aspects of their relationship, and cope with other challenges surrounding recovery from morbidity:

It [my life] has changed a lot. There has been happiness inside the house. It has been filled with happiness… When he comes and hears his child calling him, then there is happiness. At first, he would come home angry, very angry. But now he sees my child, and when you ask something from him, he does not hesitate to bring it to you. (Woman 14)

However, under strain from unwanted pregnancy, acute and chronic morbidity, social destabilisation or bereavement, it was not uncommon for women to experience the breakdown of their relationship post partum. For two couples whose baby died, their grief and a lack of understanding of the reasons for their baby’s death led to suspicion and allegations that eroded the foundations of their relationship:

After the birth, he had changed. As for care, let’s just say, I didn’t really experience any at all. After the burial, he went back home, continued working and just like that we were not on good terms as partners…he started laying accusations that I had killed the child. He said, ‘There’s no way a child can just pass on like that, he was born healthy, I am sure you killed that child. Period.’ (Woman 2)

### Theme 3: adaptation to a new normal

Women’s lives were profoundly affected by severe maternal morbidity, and for three women, the double hardship of morbidity and bereavement. Recovery during the extended postpartum period entailed adaptation to a new normal rather than a return to their pre-pregnancy and premorbidity selves. Although many women expressed gratitude to healthcare providers for the intrapartum care that likely saved their life, most received little clinical care for chronic physical, psychological or sexual health problems in the extended postpartum period. Some women were invited for postpartum visits at the hospital or primary health centre but could not afford to go back. Only one woman received a home visit from community health workers, and for most women, leaving the hospital marked the end of their medical care. Rather, women employed various coping mechanisms to navigate ongoing challenges and rebuild their lives. The duration of this adjustment period varied, with no set time frame and many women still experiencing difficulties at the time of the interview. Dimensions of adaptation to a new normal are discussed below.

#### Physical adaptation

Women described their physical recovery in the extended post partum primarily in relation to adapting to their postpartum body. This was described as regaining functioning to resume household responsibilities, and/or the absence of pain, rather than a return to their pre-pregnancy and premorbidity physical state. Women often invoked their ability to carry water again, especially after a caesarean:

Let’s say the energy I had from the beginning now it’s like my health has gone a bit down. After three months I was fine, I could carry water, and now I can carry up to ten jerricans without feeling pain. (Woman 17)

For all women, with and without severe maternal morbidity, regaining functioning took significantly longer than 42 days post partum. The duration women reported ranged from 3 months to 12 months and was typically more protracted for women who experienced severe morbidity:

I am back to normal though sometimes I get body aches, I feel my entire body aching, but I assume it’s normal. It [physical recovery] took time, around nine months. (Woman 16)

#### Psychosocial adaptation

Women’s emotional recovery after severe morbidity often meant managing persistent or prolonged postpartum mental health challenges.

If I remember the thoughts, especially the delivery situation, and the bad situation I was in with my child…. Let’s just say the thoughts are still there. They are still running in my mind, they haven’t ceased. (Woman 10)

For several women, fragmented memories of the complication or MNM event caused distress. Attempting to suppress traumatic memories was a common coping strategy:

I just erased them… I knew if I think of them continuously, they will confuse me. (Woman 3)

To manage chronic postpartum mental health challenges, some women focused on the health and needs of their child. For three women who experienced suicidal ideation during the postpartum period, their surviving children helped moderate those thoughts:

I was thinking I should just kill myself, what good do I have. But when I look at my children I say aaa when I kill myself who will remain with them. (Woman 14)

However, all women whose baby did not survive or had developmental problems expressed chronic distress. These women continued to endure grief from bereavement or child ill health:

So I’ve been asking myself why? Why did he [God] do that to me? Having stayed so long without a child just to kill my child, did he deem me unfit to take care of a child or I was to beg him to help me raise my child. That’s what has been bothering me and it stressed me out. (Woman 1)

I don’t have peace like in the beginning… the women I had given birth with have their children. I have no peace at all. I have no peace at all. (Woman 2)

Until I witness my child walking, that’s when some of these thoughts will disappear, but until then, when I look at the baby I still remember. (Woman 10)

Although women’s ongoing physical health problems, poor child health outcomes or bereavement following severe maternal morbidity were common triggers for chronic postpartum mental health problems, some women without severe morbidity also experienced symptoms of postpartum depression. Their accounts exposed significant deficiencies in postpartum mental healthcare and a lack of recognition from women’s social networks:

It took me time [to recover] and I wasn’t getting help. I’m trying to tell someone that I have a problem, but I don’t know how people treat postpartum depression. At home people think it’s a white man’s problem, you see?…There was a time I thought I would die because when this child was crying when he was small I felt like things were crying in my mind until I knock myself on the wall. (Woman 20)

For this participant, social isolation in the extended postpartum period was an adaptive strategy used to conceal ongoing mental health challenges:

I needed alone time and didn’t want to reveal anything to anyone because I was like this, and they could not understand. If anyone would ask for anything from me, I would chase them away, and shout at them, I didn’t want them to see me and say ‘That one has changed.’ (Woman 20)

#### Economic adaptation

Many women had to adapt financially to the economic shocks of out-of-pocket antepartum, intrapartum and postpartum care. The costs associated with severe morbidity were unplanned, and many couples did not have the financial capacity to withstand these costs without resorting to loans or limiting treatment:

It is something you don’t plan for so it’s a challenge. Maybe they might ask for money that you don’t have, then you are forced to ask for loans from your friends, for you to solve the problem…I wasn’t able to buy medicine because my partner was looking out for money. He would tell me, ‘buy half the dose and finish the rest the next day.’ (Woman 12)

However, among many women who experienced morbidity, especially single mothers, women highlighted the effect of motherhood and exit from the labour market on her economic recovery, more than the morbidity per se. Adapting often meant finding ways to resume paid employment:

I had to close it [grocery business] because I had no one to take care of the baby… My hope is for my baby to grow fast for me to get back into my hustles. (Woman 6)

## Discussion

Based on in-depth interviews with 20 women in Kilifi, Kenya, we provide evidence suggesting women progress through three phases of recovery after severe morbidity represented in the themes ‘loss’, ‘transition’ and ‘adaptation to a new normal’. Our grounded theory posits that this phased postpartum recovery process is (1) multidimensional; (2) cyclical across the female reproductive life course, through repeat pregnancies and recurrent episodes of maternal morbidity; and (3) protracted, far beyond 42 days post partum. These features of the recovery process support Filippi *et al*’s conceptual framework for maternal morbidity^32^ and recent reconceptualisations of postpartum vulnerability.^33 34^

Our grounded theory was developed based on the accounts of women with severe maternal morbidity, as well as three women who experienced the double hardship of severe maternal morbidity and perinatal death, and three women who experienced neither severe maternal morbidity nor perinatal death. This allowed us to test the limits of our theory and its applicability to women with different experiences. First, women whose severe morbidity coincided with the death of her baby experienced all features of ‘loss’, ‘transition’ and ‘adaptation to a new normal’, with this double burden profoundly affecting all areas of their lives. However, unlike women whose baby survived the complication, ‘adaptation’ focused on coping with grief, and recovery from morbidity became a secondary concern. Second, although based on a small sample, women without severe morbidity shared most, but not all, core experiences of ‘loss’, ‘transition’ and ‘adaptation to a new normal’, but not to the same degree. This suggests that severe maternal morbidity exacerbates and accelerates changes in women’s lives associated with childbearing more generally.

Finally, often presenting the first opportunity for women to discuss their experience after severe maternal morbidity, women’s accounts tended to focus on what they found challenging in the extended postpartum period, with few women divulging more positive experiences. Our analytical focus on these accounts should not obscure that positive and negative experiences can coexist during recovery from severe maternal morbidity.

### Loss

Women with severe maternal morbidity encountered multifaceted losses in the extended postpartum period.^35–37^ Distorted memory after the event, when exacerbated by inadequate communication from healthcare providers and the absence of her partner at key treatment stages, affected women’s understanding of their morbidity and whether postpartum symptoms were ‘normal’. Prolonged and severe loss of functioning necessitated reliance on familial support for chores, personal care and childcare for many months,^35^ mainly from their mother/mother-in-law.^38^ Partners’ postpartum support was often primarily financial,^38^ leaving some women saddened at a lack of emotional support. Finally, for some women, severe maternal morbidity precipitated a loss of autonomy over decisions related to their care and increased financial dependence on their partner.

### Transition

The experience of pregnancy, severe maternal morbidity and, in some cases, perinatal death precipitated complex transitions in women’s identity as a woman, a partner and a mother. The transition to motherhood for nulliparous or to a larger family for parous women often brought joy and happiness that at times coexisted with more negative experiences. Challenges during pregnancy, delivery and the post partum initiated transitions in family structures and in women’s relationships with her partner.^39^ Relationship breakdowns were often linked to conflicts over partner support and empathy for ongoing physical, mental and sexual health problems. Many women whose child survived the event expressed a desire to space, postpone or stop future childbearing.^40^

### Adaptation

Postpartum recovery after severe maternal morbidity required women to adapt to a new normal state, rather return to their pre-pregnancy and premorbidity selves. Physical recovery focused on regaining functioning to resume household tasks and/or the absence of pain, took all participants far longer than the 42 day postpartum period and was longer for women who had experienced severe morbidity.^6^ Psychosocial adaptation required women to deploy their own coping mechanisms^35^ to manage chronic postpartum mental health problems, and for three women with perinatal death, cope with grief.^41^ High out-of-pocket treatment costs and loss of paid employment demanded adaptive coping strategies to withstand, such as halving doses or sourcing loans.

### Implications for research, policy and practice

Women’s accounts of their lived experience of postpartum recovery after severe maternal morbidity in Kilifi, Kenya, exemplify deficiencies in the provision of intrapartum and postpartum care. First, improved communication between providers, women and their families about the morbidity experienced, treatment received and aftercare required before hospital discharge, is important for recovery and retaining trust in health services.^42^ Second, with many women unable to afford to return to the hospital for routine postpartum care,^35 39 43^ provision must be improved at the primary healthcare level with community health workers to extend its reach.^44 45^ Women who experienced severe maternal morbidity (and their children who survived) may also face chronic mental and physical health outcomes that require referral to specialist services in the extended post partum.^46 47^ With care required in some cases for many months, or even years post partum, improving outcomes for women who experienced severe morbidity is an important consideration for universal health coverage. Finally, women’s descriptions of mental health disorder after severe morbidity and/or perinatal death are consistent with the high prevalence of postpartum depression in other regions of Kenya and depict a mental healthcare system in Kilifi County that is yet to be fully fit for purpose.^48–50^ Perinatal mental health support must be integrated within maternal and child health services,^51^ with its own budget allocation^48 52^ to improve its accessibility and acceptability within communities.

### Strengths and limitations

Strengths lie in the inclusion of women from diverse backgrounds (education, age, parity, type of morbidity, live birth/perinatal death, child health), to better capture the diversity of postpartum experience. As we were unable to interview women more than once during their postpartum period, we included participants at different postpartum stages at the time of interview to better understand recovery trajectories over time. The inclusion of two additional subpopulations—of double hardship (morbidity and perinatal death) and women without severe morbidity—helped test the limits and validity of our grounded theory. Finally, our multidisciplinary and multinational team provided a broader epistemic lens.

Our study has limitations. First, our grounded theory derives from the experiences of a small group of women with a varied set of morbidities and requires validation in other populations. Further, some of the most critically ill women could not be included in our study if they were referred to higher level tertiary facilities. These women may have had worse experiences, and their recovery trajectories may differ. Second, we were unable to fully explore the effect of child developmental outcomes on women’s social, emotional and economic recovery and this requires further research. Third, due to competing workloads in the research team, five individuals conducted the interviews. Differences in interviewers’ gender, age and (non-)clinical background may have introduced some heterogeneity in interview style and women’s responses. Finally, it was not possible to analyse transcripts concurrently with data collection, and we relied on team debriefs and field memos to assess theoretical saturation.

## Conclusion

Our grounded theory reaffirms the need for research, policy and practice to reconceptualise the postpartum period as extending far beyond 42 days, to better reflect the time needed for women to recover, particularly after severe maternal morbidity.^46^ Further research is required to validate our grounded theory in other contexts and provide a richer picture of women’s postpartum recovery after severe morbidity in other settings.

### Patient and public involvement

Patients were not included in the design of this study and were not directly involved in the review of transcripts or results. However, with open-ended sections of the interview tool, and prompts for participants to discuss anything else they wanted to share, the data collected were informed by their priorities, experience, and preferences. A panel of maternal health experts, including researchers based in Kilifi, ratified the study objectives, design, and the interview schedule. Our findings were presented at the Kilifi County 2^nd^ Scientific Symposium for feedback from Kilifi-based health workers.

## Data Availability

Due to ethical and confidentiality concerns, complete transcripts cannot be published. However, all data (quotations) relevant to the study are published in the manuscript or the supplemental information. Extended quotations can be provided upon reasonable request.
