# Supplementary material for: Postpartum recovery after severe maternal morbidity in Kilifi, Kenya: a grounded theory of recovery trajectories beyond 42 days
Source: BMJ Glob Health. 2024 Jun 25;9(6):e014821. doi: 10.1136/bmjgh-2023-014821 (PMC11202725; doi:10.1136/bmjgh-2023-014821)
Supplement: Supplementary data [file bmjgh-2023-014821supp001.pdf]

Supplemental Material 1

Postpartum recovery after severe maternal morbidity in Kilifi, Kenya: A Grounded Theory of recovery trajectories beyond 42 days

Table of Contents

1. Members of the PRECISE Network 2
1. COnsolidated criteria for REporting Qualitative research (COREQ) 3
2. Criteria used to identify maternal morbidity categories 5
3. English interview guide for women with severe morbidity 8
4. Supportive quotations for super categories and themes 11

Table of Tables

Table S1 Members of the PRECISE Network 2
Table S2 COREQ Checklist 3
Table S3 Definition of potentially life-threatening conditions 5
Table S4 Definition of maternal near miss events 6
Table 5 Supportive quotations 11

1. Members of the PRECISE Network

Table S1 Members of the PRECISE Network

In-country teams	Members
THE GAMBIA: Medical Research Council Unit The Gambia at the London School of Hygiene and Tropical Medicine, Fajara	Umberto D'Alessandro, Anna Roca, Hawanatu Jah, Andrew Prentice, Melisa Martinez-Alvarez, Brahima Diallo, Abdul Sesay, Sambou Suso, Baboucarr Njie, Fatima Touray, Yahaya Idris, Fatoumata Kongira, Modou F.S. Ndure, Lawrence Gibba, Abdoulie Bah and Yorro Bah.
KENYA: Aga Khan University, Nairobi	Marleen Temmerman, Angela Koech, Patricia Okiro, Consolata Juma, Geoffrey Omuse, Grace Mwashigadi, Joseph Mutunga, Isaac Mwaniki, Moses Mukhanya and Onesmus Wanje, Marvin Ochieng and Emily Mwadime.
MOZAMBIQUE : Centro de Investigação em Saúde de Manhiça, Manhiça	Esperança Sevene, Corssino Tchavana, Salesio Macuacua, Anifa Vala, Helena Boene, Lazaro Quimice, Sonia Maculuve, Eusebio Macete, Inacio Mandomando, Carla Carrilho
**Central co-ordinating team**	
Department of Women and Children's Health, School of Life Course Sciences, Faculty of Life Sciences and Medicine, King's College London	Peter von Dadelszen, Laura A. Magee, Rachel Craik, Hiten Mistry, Marie-Laure Volvert, Thomas Mendy
Donna Russell Consulting	Donna Russell
**Co-Investigator team**	
Midlands State University, Zimbabwe	Prestige Tatenda Makanga, Liberty Makacha and Reason Mlambo
Kings College London	Lucilla Poston, Jane Sandall, Rachel Tribe, Andrew Shennan, Sophie Moore, Tatiana Salisbury and Lucy Chappell
University of Oxford	Aris Papageorghiou, Alison Noble, Rachel Craik
London School of Hygiene and Tropical Medicine	Hannah Blencowe, Veronique Filippi, Joy Lawn, Matt Silver, Joseph Waiswa and Ursula Gazeley
St George's, University of London	Judith Cartwright, Guy Whitley, Sanjeev Krishna
University of British Columbia	Marianne Vidler, Jing (Larry) Li, Jeff Bone, Mai-Lei (Maggie) Woo Kinshella, Domena Tu, Ash Sandhu, Kelly Pickerill
Imperial College London	Ben Barratt

1. COnsolidated criteria for REporting Qualitative research (COREQ)

Table S2 COREQ Checklist

**Topic**	**Item No**	**Guide Questions/Description**	**Reported on page no.**
**Domain 1: Research team and reflexivity**			
Interviewer/facilitator	1	Which author/s conducted the interview or focus group?	11
Credentials	2	What were the researcher's credentials? E.g. PhD, MD	11
Occupation	3	What was their occupation at the time of the study?	11
Gender	4	Was the researcher male or female?	33
Experience and training	5	What experience or training did the researcher have?	11
Relationship established	6	Was a relationship established prior to study commencement?	11
Participant knowledge of the interviewer	7	What did the participants know about the researcher? e.g. personal goals, reasons for doing the research	11
Interviewer characteristics	8	What characteristics were reported about the inter viewer/facilitator? e.g. Bias, assumptions, reasons and interests in the research topic	12, 34
**Domain 2: Study design**			
Methodological orientation and Theory	9	What methodological orientation was stated to underpin the study? e.g. grounded theory, discourse analysis, ethnography, phenomenology, content analysis	6
Sampling	10	How were participants selected? e.g. purposive, convenience, consecutive, snowball	2
Method of approach	11	How were participants approached? e.g. face-to-face, telephone, mail, email	11
Sample size	12	How many participants were in the study?	7
Non-participation	13	How many people refused to participate or dropped out? Reasons?	11
Setting of data collection	14	Where was the data collected? e.g. home, clinic, workplace	12
Presence of non-participants	15	Was anyone else present besides the participants and researchers?	11
Description of sample	16	What are the important characteristics of the sample? e.g. demographic data, date	10

Interview guide	1<br>7	Were questions, prompts, guides provided by the authors? Was it pilot tested?	11
Repeat interviews	18	Were repeat interviews carried out? If yes, how many?	11
Audio/visual recording	19	Did the research use audio or visual recording to collect the data?	12
Field notes	20	Were field notes made during and/or after the inter view or focus group?	13
Duration	21	What was the duration of the inter views or focus group?	11
Data saturation	22	Was data saturation discussed?	11, 34
Transcripts returned	23	Were transcripts returned to participants for comment or correction?	36
**Domain 3: analysis and findings**			
Number of data coders	24	How many data coders coded the data?	12
Description of the coding tree		Did authors provide a description of the coding tree?	12
Derivation of themes		Were themes identified in advance or derived from the data?	12
Software		What software, if applicable, was used to manage the data?	12
Participant checking		Did participants provide feedback on the findings?	36
Quotations presented		Were participant quotations presented to illustrate the themes/findings? Was each quotation identified? e.g. participant number	14-28
Data and findings consistent		Was there consistency between the data presented and the findings?	14-36
Clarity of major themes		Were major themes clearly presented in the findings?	14
Clarity of minor themes		Is there a description of diverse cases or discussion of minor themes?	14

2. Criteria used to identify maternal morbidity categories

Table S3 presents the criteria used to define potentially life-threatening conditions (PLTCs), were defined according to adapted WHO criteria[1,2] with variables included in the PRECISE study.

Table S4 presents the WHO criteria[1,2], modified criteria for low resource settings (Haydom criteria[3,4] and Tura criteria for sub-Saharan Africa[5]), and the adapted criteria we used in the qualitative study to identify maternal near miss events within PRECISE facilities

Table S3 Definition of potentially life-threatening conditions

PLTC Criteria	WHO definition	Authors' adaptation for PRECISE facilities
**1. Severe maternal complications**		
Severe postpartum haemorrhage	Genital bleeding after delivery, with at least one of the following: perceived abnormal bleeding (1000 ml or more) or any bleeding with hypotension or blood transfusion.	PPH with at least one of: use of NASG, any transfusion, systolic blood pressure below 90 mmHg or diastolic blood pressure below 40 mmHg
Severe pre-eclampsia	Persistent systolic blood pressure of 160 mmHg or more or a diastolic blood pres- sure of 110 mmHg; proteinuria of 5 g or more in 24 hours; oliguria of <400 ml in 24 hours; or HELLP syndrome or pulmonary oedema. Excludes eclampsia.	. Any recorded severe hypertension with systolic blood pressure of 160 mmHg or more or a diastolic blood pres- sure of 110 mmHg; Proteinuria of 3 g or more in 24 hours; or HELLP syndrome or pulmonary oedema. Excludes eclampsia
Eclampsia	Generalized fits in a patient without previous history of epilepsy. Includes coma in pre-eclampsia.	No change
Sepsis or severe systemic infection	Presence of fever (body temperature >38°C), a confirmed or suspected infection (e.g. chorioamnionitis, septic abortion, endometritis, pneumonia), and at least one of the following: heart rate >90, respiratory rate >20, leukopenia (white blood cells <4000), leukocytosis (white blood cells >12 000).	No change
Ruptured uterus	Rupture of uterus during labour confirmed by laparotomy.	No change
Severe complications of abortion	No guideline provided	N/A not measured
**2. Critical interventions or intensive care unit use**		
Admission to intensive care unit		Measured in the PRECISE Network but there are no ICU/high dependency unit available Rabai or Mariakani facilities.
Interventional radiology		Not measured
Laparotomy	Laparotomy (includes hysterectomy, excludes caesarean section)	No change
Use of blood products		No change

Table S4 Definition of maternal near miss events

WHO criteria	Haydom criteria	Tura criteria	Authors' adaptation for PRECISE facilities
**1. Clinical criteria**			
Acute cyanosis	Acute cyanosis	Acute cyanosis	Acute cyanosis
Gasping	Gasping	Gasping	Gasping
Respiratory rate > 40 or <6/min	Respiratory rate > 40 or <6/min	Respiratory rate > 40 or <6/min	Respiratory rate > 40 or <6/min
Shock	Shock	Shock	Shock[a]
Oliguria non-responsive to fluids or diuretics	Oliguria non-responsive to fluids or diuretics	Oliguria non-responsive to fluids or diuretics	Oliguria[b]
Failure to form clots	Failure to form clots	Failure to form clots	Failure to form clots
Loss of consciousness lasting more than 12hr	Loss of consciousness lasting more than 12hr	Loss of consciousness lasting more than 12hr	Loss of consciousness lasting more than 12hr
Cardiac arrest	Cardiac arrest	Cardiac arrest	Cardiac arrest
Stroke	Stroke	Stroke	Stroke
Uncontrollable fit/total paralysis	Uncontrollable fit/total paralysis	Uncontrollable fit/total paralysis	Uncontrollable fit/total paralysis
Jaundice in the presence of pre-eclampsia	Jaundice in the presence of pre-eclampsia	Jaundice in the presence of pre-eclampsia	Jaundice in the presence of pre-eclampsia
	Eclampsia	Eclampsia	Eclampsia
	Uterine rupture	Uterine rupture	Uterine rupture
	Sepsis or severe systemic infection	Sepsis or severe systemic infection	Sepsis or severe systemic infection
		Pulmonary oedema	Pulmonary oedema
		Sepsis or severe systemic infection	Sepsis or severe systemic infection
		Severe abortion complications	
		Severe malaria	Severe malaria
		Severe pre-eclampsia with ICU admission	Severe pre-eclampsia[c]
**2. Laboratory-based criteria**			
Oxygen saturation <90% for > 60min	Oxygen saturation <90% for > 60min	Oxygen saturation <90% for > 60min	Oxygen saturation <90%[d]
PaO2/FiO2 <200 mmHg			
Creatinine ≥ 300 μmol/l or ≥3.5 mg/dl		Creatinine ≥ 300 μmol/l or ≥3.5 mg/dl	Creatinine ≥ 300 μmol/l or ≥3.5 mg/dl[e]
Bilirubin > 100 μmol/l or > 6.0 mg/dl			
pH <7.1			
Lactate ≥5 mEq/ml			
Acute thrombocytopenia (<50,000 platelets/ml)	Acute thrombocytopenia (<50,000 platelets/ml)	Acute thrombocytopenia (<50,000 platelets/ml)	Acute thrombocytopenia (<50,000 platelets/ml)
Los of consciousness and ketoacids in urine		Loss of consciousness and ketoacids in urine	
**3. Management-based criteria**			
Use of continuous vasoactive drugs		Use of continuous vasoactive drugs	

Hysterectomy following infection or haemorrhage	Hysterectomy following infection or haemorrhage	Hysterectomy following infection or haemorrhage	Hysterectomy following infection or haemorrhage
Transfusion of ≥ 5 units of blood	Transfusion of ≥ 1 units of blood	Transfusion of ≥ 2 units of blood	Transfusion of ≥ 1 units of blood
Intubation and ventilation for ≥60min not related to anaesthesia	Intubation and ventilation for ≥60min not related to anaesthesia	Intubation and ventilation for ≥60min not related to anaesthesia	Intubation and ventilation for ≥60min not related to anaesthesia
Dialysis for acute renal failure			
Cardio-pulmonary resuscitation	Cardio-pulmonary resuscitation	Cardio-pulmonary resuscitation	Cardio-pulmonary resuscitation
		Laparotomy other than caesarean section	Laparotomy other than caesarean section
	Admission to intensive care unit		Admission to intensive care unit[f]

[a] Shock defined as any case meeting either of the following criteria:
(i) systolic BP < 90 mmHg with heart rate >120 with IV fluids;
(ii) (iii) systolic BP < 90 mmHg or diastolic < 40 mmHg with heart rate >90 or respiratory rate > 20 or oliguria <30ml/4hr.
[b] Use of diuretics not specified
[c] ICU admission for severe pre-eclampsia not specified because most PRECISE facilities do not have ICU or high dependency units.
[d] Duration of oxygen saturation below 90% not specified
[e] Creatinine measurement not universally available.
[f] Measured in the PRECISE Network but there are no ICU/high dependency unit available Rabai or Mariakani facilities.

3. English interview guide for women with severe morbidity

Interview introductory greetings

[Interviewer to introduce themselves, and briefly remind the participant of the purpose of the study, estimatedduration, and remind the participant of informed consent procedures]

[For women who experienced a stillbirth or neonatal death only:]
Our sincere condolences to you and your family for the loss of your baby.

How are you, and [if applicable] how is your baby?"

Recollection of pregnancy and delivery

[Interviewer to clarify whether the woman has had another pregnancy since the pregnancy where she experienced a complication. Clarify that the questions you will ask refer to the pregnancy with complications]

In your own words, could you tell me about your last pregnancy and how you felt?
Potential probes if not mentioned in conversation:

- Can you tell me about when you found out you were pregnant and how you felt? Was the pregnancyplanned?
- How did you feel during your pregnancy?
- How did you feel during delivery?
- Did you have any problems during pregnancy?
- When did you first learn that there was a problem?
- Did the doctors explain to you what the problem was? Did you understand what was happening at thetime?
- What treatment did you receive?
- How long did you spend in the hospital before being discharged?

Looking back now at your experience of pregnancy and delivery, how do you feel when you think aboutthat time?

- How have your feelings about what happened changed over time?

Aftermath and Recovery from Maternal Morbidity

Now I'd like to ask you about how you have been since the pregnancy/delivery. *Would you like to take abreak, or are you okay to continue?*

A. Events following pregnancy/delivery

In your own time, and in your own words, can you tell me what happened once you came home fromthe hospital after the pregnancy had ended/ after the birth of your child?
Potential probes:

- What happened soon after you got home?
- How did you feel to be home?

B. Postpartum care and support

Can you tell me about the care you received from health service providers since the birth [since the lossof your baby] and in the weeks and months that have followed?
Potential probes:

- This could be care you received from health services, Community Health Workers (CHW), or anyother provider of postpartum care.
- For how long after the birth did you receive this care? Did you feel this was long enough?
- What could the health workers have done better to support you and your partner during thepostpartum period?
- Are there any other services you wish had been offered to you that weren't?

Now can you tell me about the support you have received since the birth [and since the loss of yourbaby] and in the weeks and months that have followed?
Potential probes:

- Who supported you? This could be support from your partner, other family members, friends, amember of the community, traditional birth attendants, neighbours, or a member of your church/mosque.
- Support could be in the form of help with housework and household responsibilities, financialsupport, or social and emotional support.
- What support did you receive from your husband? What about from your mother? And mother-in-law?
- For how long after the birth did you receive this support? Was this long enough? How did the supportyou were offered change over time?
- Are there any types of help or support you wish had been offered to you that wasn't?
- Have you felt people around you have understood what you went through?

Can you tell me about how your life has changed since your last pregnancy and with your new baby/ following the loss of your baby?

Physical recovery: How have you felt physically since the delivery / since the loss of your baby?
Potential probes:

- You gave birth in [INSERT MONTH/YEAR]. Could you tell me about your physical recovery in theweeks and months that followed? What does it mean to you to feel physically recovered from the pregnancy and birth? How long did it take for you to feel physically recovered?
- How has how you have felt physically changed over time?
- If you feel like you have not yet physically recovered, in what way? How does this make you feel?

Emotional recovery: How have you felt emotionally since the delivery / since the loss of your baby?

- Could you tell me about your emotional recovery in the weeks and months that followed?
- In the weeks and months that have followed the delivery, what emotions have you felt? Have you felthappy? Sad? Worried? Anxious? In control? Are there any other emotions you felt?
- How has how you have felt emotionally changed over time?
- If you feel like you have not yet emotionally recovered, in what way? How does this make you feel?
- Has the complication affected your self-esteem, identity, and body image, and if so, how?

Social recovery: How have you felt emotionally since the delivery / since the loss of your baby?

- Could you tell me about the social impact the complication has had on you in the weeks and monthsthat followed?
- Could you tell me about how the complication has affected your relationship with your partner?
- How has it affected your sex life? When did you feel ready to have sex again?
- How about any other members of your family?

Economic recovery: How have you been financially since the delivery / since the loss of your baby?

- Could you tell me about the economic impact the complication has had on you in the weeks andmonths that followed?

Plans for the future

What are your hopes and plans for the future?

- *[If she mentions only hopes, then probe plans. If she only mentions plans, probe hopes.]*
- Potential Probes:
- How has your experience at pregnancy/delivery and the complication changed your hopes or plans forthe future?
- Have you been pregnant again? How does the thought of a future pregnancy make you feel?
- Have your feelings towards a future pregnancy changed since your experience, and if so, how?
- How has the complication has affected your partner’s hopes and plans for the future? In what way?

Final remarks

- What advice would you have for other women who have experienced something similar to you?
- Do you have anything to add about your experience which perhaps I haven’t asked you about?
- Do you have any questions for me?

I would like to thank you very much for your time on behalf of Aga Khan University, PRECISE Study team.Thank you for sharing your story with us. We understand that today may have brought up difficult feelings and memories for you. If you would like to receive counselling support, please let me know and I will link you to the facility counsellor. [IF APPLICABLE]: Please accept my deepest condolences again for the death of your baby.

4. Supportive quotations for super categories and themes

Table S5 Supportive quotations

Theme	Super category	Participant	Quotation	Interpretation
**Loss**	**1a. Of understanding**	**Woman 13**	*"I went back because I was in a lot of pain even walking. I was walking slowly and when I walk I just feel pain. I failed to understand whether it is giving birth or what is it? Because even if it is injection… what is the biggest problem here?"*	Poor understanding of the PLTC or MNM event and their expected recovery trajectory contributed to women's ongoing mental and physical pain
		**Woman 12**	*They didn't explain the reason [for the operation] because myself I was awake but didn't understand much, could not even remember the pin number of my phone".*	Some women saw their loss of cognitive functioning during the event as the reason why health care workers did not sufficiently explain what was happening.
		**Woman 9**	*[So did the doctors explain the problem? What did they say the problem was?]* *"Because that time I came, I was semi-conscious, and was brought here directly. I was just hearing the doctor saying let's take her for scanning and she will be taken to the maternity ward. That time I was with my friend and we went to the ward so I didn't know if my friend was told."*	
		**Woman 5**	*[Now they just told you had a problem, but did they explain to you what the problem was?] "They just said during the time of delivery it's when I started convulsing"/ [Ooh, did they tell you the cause of the convulsions?] "No".*	Health care worker explanation was often inadequate.
		**Woman 12**	*Some of these things you see can surprise you, is it discrimination or what?" [What made you feel you were being treated unfairly?] Now see, you see these husbands of ours. I can't even blame my husband's people being that I'm his wife. Even if there's no transport vehicles, is the distance to Mariakani too long? You can do whatever it takes to get there? He didn't come" & "He took like a whole month… He called but that couldn't tell the importance of a partner. If I was a man and my wife was pregnant, and my wife was going through a hard time and struggling then I wouldn't stay far away. He is a big*	The absence of her partner during pregnancy, the morbidity event and/or during the postpartum period contributed to impacted women emotionally and contributed to their poor understanding of the morbidity they experienced.

			problem. I gave birth at 10 and stayed unconscious til two in the afternoon. He didn't even worry about the wellbeing of his wife. It's not good, I felt bad about it".	
		**Woman 2**	*"They didn't explain. What they told me was that my temperature was high. Upon giving birth, they never explained what had transpired I was just discharged and I went home and haven't been called."*	Poor understanding about the event also affected women whose morbidity coincided with neonatal death or stillbirth, and their understanding of the cause of death. Health care workers did not sufficiently explain what occurred and why the baby died.
	**1b. Of functioning**	**Woman 6**	*"Just after delivery the problem became sleeping a lot. Sleeping a lot that I couldn't understand myself, even if someone talks to me I won't hear."*	Loss of functioning contributed to a feeling of disconnection from women's sense of self.
		**Woman 20**	*"I have tried to tell my husband but he has never undergone experiences women go through so when you tell him he thinks that you are exhausted by house chores and the stress of laying that is why you are tired, but he won't understand the pain you feel"*	Some women felt there was a lack of empathy and understanding from their partner about the severe morbidity she experienced and their loss of functioning in the postpartum period.
		**Woman 15**	*"The support that I wish for and I am now still wishing for was that of my partner that I have never had. I want even today what we call love [[F; mmhm]] I don't have that thing, so I feel so lonely though my brother helps me".*<br><br>*"I don't know what happened because we used to love each other madly but it reached a time where he didn't call me, and he wasn't doing anything I needed. I would tell him I want to buy maybe medicine that doctor has prescribed medicine to boost blood, fruits or something, he said 'okay I will send you', but in one week I would not get the money or the medicine".*	Some women expressed sadness at the lack of support from her partner in the postpartum period.

		**Woman 11**	*"She [her mother-in-law] used to prepare hot water for me, bathe me, cook for me, bathe my baby. And she helped me to take the medicine I was given at the hospital".*	Personal care was usually provided by either women's mother or mother-in-law.
		**Woman 8**	*"My husband used to help me in bathing myself, he used to carry water to the bathroom and help bathe me"*	Care from the husband with bathing was a rare exception.
		**Woman 4**	*"My sister helped with like cooking, washing clothes, and my husband let's say on the side of holding me he was supportive. There were no problems, he was very supportive,"*	Some women reported that their husbands provided adequate emotional support.
		**Woman 10**	*"My mother is deceased, my mother died that day I gave birth to this child and my mother in law lives far away from here, so it was me, my husband and my childen"*	In exceptional cases where personal care derived predominantly from the husband, women's mother or mother-in-law were not alive or close by to provide support.
		**Woman 3**	*"There was a time where there was no happiness in the household, I don't know why. There was a time where they started competing for the chores, do it, do it. They did not want [to do the chores]"*	Women's loss of functioning led to conflict within some households, with disputes about who would assume responsibility for chores.
		**Woman 14**	*"Eeeh he used to get angry, his work was just to get angry… It's that, not meeting up with other people outside."*	In some relationships, women's loss of functioning also led to conflict. This woman's husband was angry at her postpartum social isolation.
		**Woman 18**	*"They used to go walking and I couldn't. I was indoors 24/7 just in with the baby."*	Loss of physical functioning and an inability to participate in activities led to social isolation in the postpartum period.

	**1c. Of autonomy**	**Woman 8**	*"I went back home when I was seven months [postpartum]. So my husband told me to go to my mother in law's since he used to go to work."*	A reliance on women's mother-in-law for household support was sometimes against women's wishes
		**Woman 16**	*"Sometimes I think and say, "why did I even get pregnant". Back then did you treat me like this or it's now that I have become a mother you are treating me this way. So I think a lot and that is why this thought of going back to school comes to me. I got pregnant because I did not have any certificate, I am just a housewife, and that is why you are treating me this way. Wait until I go back to school. The day I have my certificate you will respect me."*	Disruption to women's education, and loss of autonomy to decide their own career path, affected adolescent mothers, with or without severe morbidity.
		**Woman 16**	*"Once I got pregnant, I had to stop [working]. I was used to getting money and that was my money. I used to decide what to do, now if you go there you sit to be given money, and then it is budgeted. Now there were things I could not accomplish, like I used to send my mum some money."*	Pregnancy and postpartum recovery initiated a loss of financial autonomy and greater reliance on her husband for financial needs.
**Transition**	**2a. In identity**	**Woman 19**	*"[After delivery, how did you feel when you saw the baby?] I was happy now being a mother (laughter)"*	Some nulliparous women expressed happiness at their transition to motherhood.
		**Woman 14**	*"You see at home where I have been married to when they heard I lost the first pregnancy they started to say, "that is not a wife she is just playing she is playing with your mind" so when I got this one even if it really hurt me but when I got it even them themselves told me "now come home we know now that you are a wife."*	Pregnancy initiated complex transitions in women's identity as a wife. Without producing a healthy live baby, community members perceived women to be unqualified as a wife.
	**2b. In relationships**	**Woman 4**	*"My husband was patient enough. It's me who said now is enough is enough, but he was okay. He was caring, he is not among the ones who says "oooh we have stayed [without sex] for long" cause I think we had finished a month and some weeks"*	Husbands who were patient and understanding of their wives' decision not to have sex after the PLTC or MNM event were rare. This woman's partner was an outlier in partner's responses to delayed sexual activity.
		**Woman 16**	*"For me after seven months, six months actually, I was not having sex again with him for experiencing pain and I was suffering a lot. I stayed like that until the baby was six or*	Some women delayed the resumption of sexual activity for many months in the

			seven months and then I resumed sex, and normally it would be painful… He told me when I visit a clinic I explain myself since it was not normal. Normally during sex one should not experinece pain. I have never enjoyed, I just hear people say having sex is pleasant. For me it is just painful."	postpartum due to chronic pain.
		**Woman 14**	*"Something that surprised me is pain, I did not know where the pain is coming from, another pain eee now that happiness wasn't happiness again… The first time he told me I am pretending. There is no enjoyment."*	One partner displayed coercive behaviour to try to resume sex with allegations his wife was pretending that sex was causing her pain.
		**Woman 12**	*"Eee I want to take a break… because I have passed through a lot… For about five years. [Why?] Let's say it's also healthy. For example, I gave birth with all problems. If after one year I got pregnant, I would have given birth again and at three years my baby would have had a younger sibling, and then the same cycle every year again, wouldn't it be harmful? That's the reason I decided to take a break".*	Motivated by a need to regain strength after the complication, many women expressed a desire to space or postpone future childbearing.
		**Woman 8**	*"My hope is even when I become pregnant again, I should never go through what I went through [before]".*	The PLTC or MNM event women experienced often affected women's attitudes towards a future pregnancy.
		**Woman 10**	*"Yes they [future fertility plans] have changed… Mmmhh I thought I would carry this and the other, mmmh but I said let me stop at that, that's enough"*	
		**Woman 15**	*"It's only just pain because even the one who told me I killed my child I decided to leave him I didn't keep it in my mind I left him to speak like a crazy person… My partner said I killed the baby."*	Two husbands blamed their wife for the perinatal death, contributing to a breakdown of the relationship.
**Adaptation**	**Postpartum care**	**Woman 10**	*"Aaaah there's no care [in the postpartum]. Mmmm because when you get discharged you are removed."*	For many women, hospital discharge was described as the point at which their medical care ended.
	**3a. Physical**	**Woman 7**	*"From six months onwards, I felt good. I could carry a ten litre jerrican of water. It was good progress."*	Women's ability to carry water again was a common barometer of having physically recovered from the severe maternal morbidity.
		**Woman 18**	*"I was good in two weeks, I could even carry water… 20 litres. I myself I could do everything [by two weeks], it was just that they didn't want me to… They were saying I*	

			hadn't fully recovered when for me I was feeling totally healed. They said if I do the chores then later on I'd have pain."	
		**Woman 10**	*"Mmmh after three months I was fine. Mmmm I used to carry water"*	
		**Woman 1**	*"My body was fine because after three months I was told to rest for three months, not to do hard work, not to lift heavy things, when I obeyed the conditions, my scar healed."*	Women's recovery in their ability to carry water and other heavy objects was associated with delivery caesarean section, though some women with vaginal births also expressed recovery in these terms.
	**3b. Psychological**	**Woman 13**	*"You see that difficult situation in the six months, at the start when that situation was reducing, I continued to feel happiness in my body."*	Progress with physical recovery in the postpartum period contributed to women's emotional wellbeing.
		**Woman 18**	*"I had gone through all the challenges and now my baby is healthy, it was good, and I felt excited".*	The health of her baby brought women happiness in the postpartum period.
		**Woman 13**	*"I got my child in full health, but I am going through a trying time from a problem I don't understand."*	For women whose baby survived, concern for the child's health often outweighed concerns about their own recovery.
		**Woman 14**	*"The one who did for me scanning told me "if the child has reached eight months and hasn't turned it might die in the stomach at the time of giving birth" so it was time to give birth and I said maybe it has died, but the moment I saw him and the moment I reached home I felt happiness because the devil was ashamed what they were talking about is different and they changed their words"*	Women's emotional recovery was mediated by the health of her child. For this participant, the survival of her child against the warnings of health care workers that she might lose the pregnancy brought happiness.
		**Woman 10**	*"It has affected because when I go to church and witness other babies walking, those who we gave birth with I see their children walking and others sitting and others are*	Poor health of her child affected women's emotional recovery from the complication.

			standing, then I look at my child and ask God what I did wrong?"	
		**Woman 1**	*"I cried for my son for a whole year."*	Bereavement caused ongoing pain.
	**3c. Economic**	**Woman 12**	*"I was able to buy medicine because my partner was looking out for money. He would send me, "buy half the dose and finish the rest the next day". That was the situation. We could not afford full doses, I would buy doses for two or three days, until I got well".*	At times, treatment for chronic conditions following the pregnancy complication in the extended postpartum period was unaffordable, and women were forced to delay or limit their treatment.
		**Woman 17**	*My life has changed because initially I could step out and go and do any kind of job or say let me today go to work because someone has called me saying "come there is something" and I do. But now it's like my life has become a litter harder, because now who will hire you and the baby? No one. You just do a little bit of laundry, I clean dishes, I do some cleaning, and be given the little I will be given, that's all."*	Among many women who experienced morbidity, women highlighted the effect of (single) motherhood and exit from the labour market on her economic recovery, more than the complication *per se*.

References

1. Say L, Souza JP, Pattinson RC. Maternal near miss – towards a standard tool for monitoring quality of maternal health care. Best Pract Res Clin Obstet Gynaecol. 2009 Jun 1;23(3):287–96.

2. WHO. Evaluating the quality of care for severe pregnancy complications: the WHO near-miss approach for maternal health. 2011.

3. Nelissen EJ, Mduma E, Ersdal HL, Evjen-Olsen B, van Roosmalen JJ, Stekelenburg J. Maternal near miss and mortality in a rural referral hospital in northern Tanzania: a cross-sectional study. BMC Pregnancy Childbirth. 2013 Dec;13(1):141.

4. Nelissen E, Mduma E, Broerse J, Ersdal H, Evjen-Olsen B, van Roosmalen J, et al. Applicability of the WHO Maternal Near Miss Criteria in a Low-Resource Setting. Young RC, editor. PLoS ONE. 2013 Apr 16;8(4):e61248.

5. Tura AK, Stekelenburg J, Scherjon SA, Zwart J, van den Akker T, van Roosmalen J, et al. Adaptation of the WHO maternal near miss tool for use in sub-Saharan Africa: an International Delphi study. BMC Pregnancy Childbirth. 2017;17(1):445.
