# Supplementary material for: Postpartum recovery after severe maternal morbidity in Kilifi, Kenya: a grounded theory of recovery trajectories beyond 42 days
Source: BMJ Glob Health. 2024 Jun 25;9(6):e014821. doi: 10.1136/bmjgh-2023-014821 (PMC11202725; doi:10.1136/bmjgh-2023-014821)
Supplement: Supplementary data [file bmjgh-2023-014821supp002.pdf]

Supplemental Material 2

Postpartum recovery after severe maternal morbidity: a Grounded Theory of recovery beyond 42 days

Authors

Ursula Gazeley[1,2]*, Marvine Caren Ochieng[3], Onesmus Wanje[3], Angela Koech[3,4], Grace Mwashigadi[3], Nathan Barreh[3], Alice Mnyazi Kombo[3], Mwanajuma Bakari[3], Grace Maitha[3], Sergio A. Silverio[5,6], Marleen Temmerman[3,4], Laura A. Magee[5,7], Peter von Dadelszen[5,7], Veronique Filippi[1], and the PRECISE Network[8].

Affiliations

[1] Department of Infectious Disease Epidemiology, London School of Hygiene and Tropical Medicine, London, UK

[2] Population Studies Group, London School of Hygiene and Tropical Medicine, London, UK

[3] Centre of Excellence in Women and Child Health, Aga Khan University, Nairobi, Kenya

[4] Department of Obstetrics and Gynaecology, Aga Khan University, Nairobi, Kenya

[5] Department of Women and Children's Health, School of Life Course and Population Sciences, King's College London, London, UK

[6] School of Psychology, Faculty of Health, Liverpool John Moores University, Liverpool, UK

[7] Institute of Women and Children's Health, King's College London, UK

[8] The full list of PRECISE members is in the supplementary material (Table S1)

* Corresponding author

London School of Hygiene and Tropical Medicine, London, WC1E 7HT, ursula.gazeley@lshtm.ac.uk

Author reflexivity statement

Area	Question	Answer
Study conceptualisation	How does this study address local research and policy priorities?	No existing research has studied women's recovery after severe morbidity in Kilifi, Kenya. As local context is so important to women's experience of morbidity and recovery, this research was designed to address a critical evidence gap: the complex needs of women recovering from severe maternal morbidity in Kilifi County, so that services can be planned.<br><br>Postnatal care was raised at the Kilifi County Scientific Symposium, where our work (presented by MCO) won the award for the best poster presentation. This recognition from policy makers and healthcare workers working in Kilifi County is an indication of the importance of this topic locally.
	How were local researchers involved in study design?	The initial idea for the study was suggested by the lead author (UG) who is doing a PhD at LSHTM in the form of a concept note and discussed with leads in Kenya who suggested changes to the sampling strategy, participant recruitment, and interview tool (AK, MT).<br><br>An introductory session before data collection began was held with all Kenyan team members to refine the study design. This provided an opportunity for members of the research team to suggest further changes to the study design. Substantive changes were made to the interview tool and participant recruitment plans.
Research management	How has funding been used to support the local research team(s)?	This qualitative study was supported by the UKRI Economic and Social Research Council as the funders of UG's PhD studentship (ES/P000592/1), as well as a doctoral travelling scholarship awarded to UG from LSHTM.<br><br>This funding was used to support the local research team with regards to recruitment, reimbursement for time and travel, training (including qualitative analysis group training), and costs of consultants (transcription and translation).<br><br>The PRECISE Network was funded by UKRI GCRF Award (MR/P027938/1) and a NIHR–Wellcome Partnership for Global Health Research Collaborative Award (217123/Z/19/Z). This has provided the salaries for all Kenyan named co-authors. The

Area	Question	Answer
		UKRI award is a capacity-building grant designed to develop scientific research capacity in Africa.
Data acquisition and<br>analysis	How are research staff who conducted data collection acknowledged?	All research staff who conducted the qualitative data collection are co-authors of this paper (MCO, NB, AMK, MB, and GMa). Kenyan research staff who were involved in the logistics of sample recruitment and data collection organisation are also co-authors (OW, AK, GMw).<br>Other members of the PRECISE Network who conducted the PRECISE data collection, which was used as a sampling frame to identify women with severe morbidity for this qualitative study, are also authors in the PRECISE Network. (The full author list is available in Table S1.)
	How have members of the research partnership been provided with access to study data?	All the study data is held at Aga Khan University, Kenya, with copies shared to LSHTM.<br>Three members of the research team who conducted the analysis (UG, OW and MCO) had access to the full transcripts. All Kenyan members of the research team were involved in a full-day qualitative analysis workshop, where one transcript analysed together.
	How were data used to develop analytical skills within the partnership?	This study contributed to the analytical capacity-building within the partnership. The Kenyan research team had a qualitative method training session led by the first author (UG). This training session was designed to build team members’ experience with the qualitative data after it has been collected – including transcription processes, data coding, analysis, interpretation, and results write-up.<br>Further training on qualitative data interpretation and results write-up was provided during virtual hands-on sessions by UG to two Kenyan early career researchers (MCO, OW). UG also provided mentorship on proposal writing and conference submission to MCO.
Data interpretation	How have research partners collaborated in interpreting study data?	All named co-authors provided critical interpretation of the results upon review of the manuscript. Interpretation discussions were held between the three authors involved in data analysis (UG, MCO, OW) and with UG’s supervisory team (VF, LAM, PvD).

Area	Question	Answer
		Where differences in interpretation emerged, the interpretation of Kenyan members of the research team was prioritised, given their greater contextual exposure and understanding.
Drafting and revising<br>for intellectual content	How were research partners supported to develop writing skills?	UG (an early career researcher) drafted the manuscript. The manuscript was revised critically for intellectual content by Kenyan early career researchers. For some members of the research team, editing this article was one of their first exposures to scientific journal article writing.<br>The research findings were also presented by a Kenyan early career researcher (MCO) at the Kilifi County 2nd Scientific Symposium poster session, which helped to develop her science communication skills to summarise the research findings for a poster presentation.
	How will research products be shared to address local needs?	Our findings highlighted areas for improvement in postpartum physical and mental health care in Kilifi County. These results will be included in future PRECISE Network dissemination which includes healthcare workers and local community members.<br>In addition, these policy recommendations were presented at the Kilifi County 2nd Scientific Symposium. This was a county-level meeting: most of the guests were healthcare workers, healthcare managers, policy makers from Kilifi County Department of Health, and several NGOs and research institutions working in Kilifi County.
Authorship	How is the leadership, contribution, and ownership of this work by LMIC researchers recognised within the authorship?	9 of the 14 co-authors in the research team are affiliated with the Aga Khan University in Kenya (MCO, OW, AK, GMw, NB, MK, AMK, GMa, MT). Eight of these authors are Kenyan nationals. Three Kenyan early career researchers, who are second, third and fourth authors on the paper, respectively (MO, OW, AK), played substantial roles in the logistics, participant recruitment, data collection, analysis, interpretation and manuscript revision.
	How have early career researchers across the partnership been included within the authorship team?	Nine co-authors in the research team, including the first author, are ECRs (UG, MCO, OW, GMw, NB, MK, AMK, GMa, and AK).

Area	Question	Answer
	How has gender balance been addressed within the authorship?	10 of the 14 co-authors in the research team are female, including the first (UG) and senior author (VF).
Training	How has the project contributed to training of LMIC researchers?	This project has contributed to the qualitative methods training, scientific journal writing skills, and conference presentation skills of Kenyan researchers (see above).
Infrastructure	How has the project contributed to improvements in local infrastructure?	Some of the funds from this qualitative project will contribute to minor repairs of the PRECISE study offices. Further, the PRECISE Network, within which this sub-study is embedded, has contributed to substantial improvements in local infrastructure. New study office buildings were commissioned, and new laboratory equipment was purchased. More detail about the PRECISE Network can be found here.
Governance	What safeguarding procedures were used to protect local study participants and researchers?	The research team used distress protocols to identify participants who experienced negative emotional responses to participation in the study. Participants who raised concerns over their mental or physical health during the interview were provided with referral information for follow-up care. We have formally engaged a psychologist to assist in providing mental health support to both participants and staff. Participants of this study (especially those who lost a child) have received individual and/or group counselling. This is ongoing.<br><br>To safeguard the wellbeing of the research team, we held daily debriefs to offload after potentially triggering interviews. We also had two debrief sessions during data collection with a trained psychologist to offload and process after emotionally difficult interviews.
